# Comparison of two immunotoxins against DLL3 receptor; as an inhibitor for small cell lung cancer

**DOI:** 10.3389/fmolb.2025.1506768

**Published:** 2025-03-19

**Authors:** Mohammad Hossein Ataee, Seyed Ali Mirhosseini, Reza Mirnejad, Hamideh Mahmoodzadeh Hosseini, Jafar Amani

**Affiliations:** ^1^ Student Research committee, Baqiyatallah University of medical Sciences, Tehran, Iran; ^2^ Applied Microbiology Research Center, Biomedicine Technologies Institute, Baqiyatallah University of Medical Sciences, Tehran, Iran; ^3^ Molecular Biology Research Center, Biomedicine Technologies Institute, Baqiyatallah University of Medical Sciences, Tehran, Iran

**Keywords:** DLL3, SCLC, rovalpituzumab, immunotoxin, Rova-GrB, Rova-Typh, typhoid toxin

## Abstract

Despite the efforts of researchers to develop new treatments for small cell lung cancer (SCLC), achieving effective treatment has not yet happened. Targeted therapy utilizing delta-like ligand 3 (DLL3), which is highly expressed in SCLC patients, holds promise as a potential solution. Immunotoxins, consisting of bacterial toxins from the ADP-ribosyl transferase toxin family have shown effectiveness in targeting cancer cells. In this study, we investigated the binding ability, cytotoxicity, apoptosis induction rate, and permeability of two immunotoxins based on the rovalpituzumab antibody. The binding ability of immunotoxins to the receptor was performed by the Cell-ELISA method. Following this, the cell viability of cancer and normal cells immunotoxins was evaluated using the MTT assay. The ability to induce apoptosis and the penetration of immunotoxins was assessed by flow cytometry and Western blotting method. The binding ability of the immunotoxin Rova-Typh to the DLL3 receptor was higher compared to the immunotoxin Rova-GrB. The cell viability of A549 cancer cells treated with immunotoxins showed IC50 concentrations of 338 and 734 nM for immunotoxins Rova-GrB and Rova-Typh, respectively. The induction of apoptosis by immunotoxin Rova-Typh was greater compared to immunotoxin Rova-GrB. The designed immunotoxins in prokaryotic hosts exhibit good anticancer performance in A549 lung cancer cells. Additionally, the bacterial toxin-based immunotoxin has a greater ability to induce apoptosis compared to human enzymes and can be considered as a therapeutic option for SCLC cancer.

## 1 Introduction

Lung cancer, reported as the leading cause of cancer incidence and mortality among men in the latest Global Cancer Statistics report (Sung et al., 2021), remains a formidable challenge. The SCLC, comprising 13%–15% of all lung cancer cases, presents unique hurdles due to its aggressive nature, rapid metastasis, frequent recurrence, and the absence of effective new treatments. Cancer initiation is a multifaceted process involving both genetic and environmental factors. The aggressive nature of small cell lung cancer (SCLC) demands a thorough understanding of its molecular underpinnings (Bhat AS et al., 2024). This enigmatic cancer has earned the name “graveyard of drug development” in the oncology field (Oronsky et al., 2017; Travis et al., 2015).

The use of immunotoxins in cancer treatment has seen both advances and setbacks since its inception in the 1980s. Recent advancements, such as recombinant protein production methods, have allowed for the precise targeting of cancer cells while minimizing off-target toxicity and autoantibody production. Notably, immunotoxins based on *Pseudomonas* exotoxin A have been extensively explored in the context of various cancers, including lung, breast, bladder, esophageal, gastric, and pancreatic cancer (Pai et al., 1996) RG7787 against NSCLC (Non-small cell lung cancer) (Alewine et al., 2014) LMB-100 against Mesothelioma and pancreatic cancer (Alewine et al., 2020) and hGC33-PE38 against Small cell lung cancer (SCLC) & Pancreatic cell line (Rodakowska et al., 2021). Despite the promising results, only Moxetumomab pasudotox has been approved as the *pseudomonas* exotoxin A-based immunotoxins for the treatment of hairy cell leukemia. Research is needed to use other bacterial toxins in the structure of immunotoxins for more effective treatment.

While several common biomarkers are associated with lung cancer, including EGFR, ALK, KRAS, ROS1, HER2, and others (Villalobos and Wistuba, 2017), the high expression of DLL3 in SCLC has emerged as a promising new target for therapeutic intervention (Sharma et al., 2017). Rudin et al. (2017) demonstrated the potential of Rova-T (rovalpituzumab teserine), which specifically targets Delta-like canonical Notch ligand 3 (DLL3), a unique biomarker of SCLC (Rudin et al., 2017). Overexpression of DLL3, an inhibitor of the Notch signaling pathway, has fueled interest in targeting this ligand for drug development, resulting in the exploration of agents like Rova-T, AMG 757, AMG 119, DLL3-targeted bispecific antibodies, and CAR-modified T cells (Owonikoko et al., 2020; Byers et al., 2019).

The use of Near-infrared photo immunotherapy to target DLL3 and the application of combination of radioisotopes and DLL3-targeted therapies, represents innovative approaches in the treatment of SCLC. New therapeutic strategies targeting DLL3 have shown promising results. However, the emergence of drug resistance, side effects including hematological, infectious, and allergic complications, as well as impacts on the immune system, pose significant challenges for these new treatments, leading to a reduction in the patient’s immune response (Ding and Yeong, 2024).

The design of immunotoxin gene constructs based on bacterial and human toxins, along with the evaluation of their anticancer effects, could serve as another effective therapeutic approach targeting DLL3 (Ding and Yeong, 2024).

The recombinant immunotoxins produced in our previous research were designed, and simple bioinformatics analyses were performed on them. Subsequently, synthetic genes were expressed in the *E. coli* BL21DE3 host, purified using nickel columns, confirmed by Western blotting, and their secondary structure was examined by CD method. Our research results demonstrated that both immunotoxins have the capability to be evaluated in cellular assay experiments (Ataee et al., 2022). In the present study, we target DLL3 using the single-chain fragment variable (scFv) of the rovalpituzumab antibody, coupled with a typhoid toxin or a granzyme B to form an immunotoxin. These immunotoxins, Rova-GrB (rovalpituzumab-granzyme B) and Rova-Typh (rovalpituzumab-typhoid), are designed to enter cancer cells and release toxins, namely, typhoid toxin and granzyme B, to induce cancer cell destruction.

The primary objective of our research is to evaluate and compare the anticancer effects of two immunotoxins: one based on bacterial toxin and the other on human enzyme in a lung cancer cell line. To facilitate clarity in naming conventions, we refer to the scFv of the rovalpituzumab antibody as “scFv of Rova,” granzyme B as “GrB,” and PltA of typhoid toxin as “typhoid toxin.”

## 2 Materials and methods

### 2.1 Expression, purification & confirmation of recombinant immunotoxins

**FIGURE 1:**
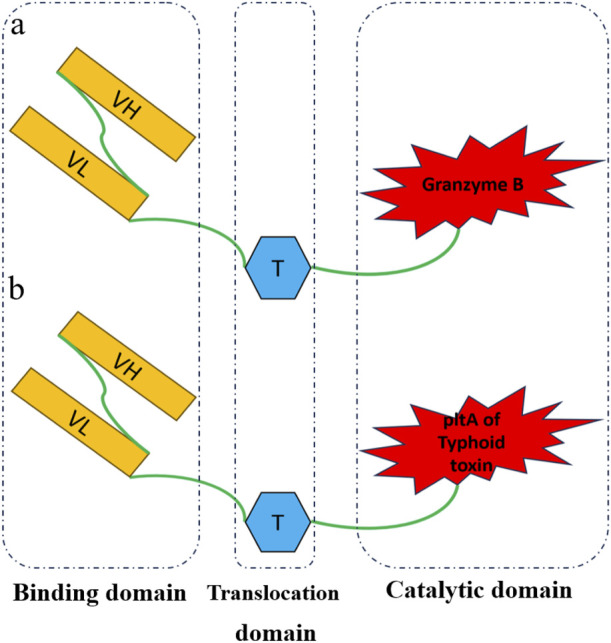
Schematic diagram of recombinant immunotoxins. Rova_GrB **(A)** and Rova-Typh **(B)** includes the single-chain variable fragment of the Rovalpitzumab antibody (binding domain), the translocation domain of exotoxin A of *Pseudomonas* (translocation domain), Granzyme B and PltA of typhoid toxin (catalytic domain), respectively.

**FIGURE 2:**
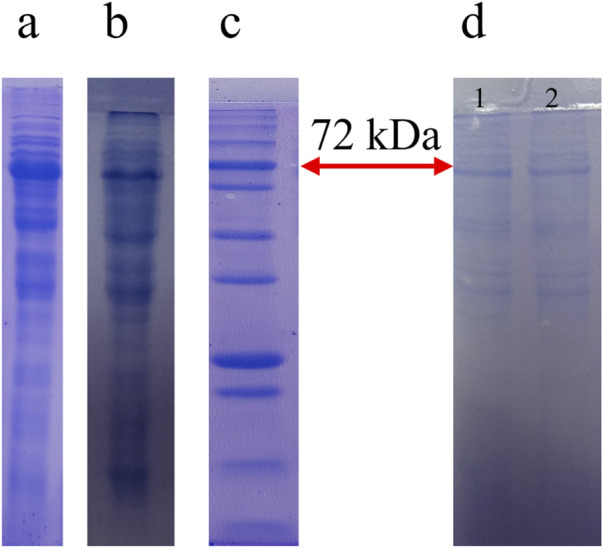
The Result of expression, purification of the Rova-GrB and Rova-Typh. Expression of Rova-GrB **(A)** and Rova-Typh **(B)** after induction by 1 mM IPTG at 24 h, protein marker, PM1700, SMOBIO Technology **(C)** and protein purification of immunotoxins **(D)**: (1) Rova-GrB purified, (2) Rova-Typh purified.

In the previous research, two immunotoxins named Rova-GrB and Rova-Typh were designed (Figure 1), bioinformatic analysis was done, the recombinant protein was cloned and expressed in E.coli BL21DE3 (Figure 2), and confirmed by Western blot using mouse anti-His tag antibody (Ataee et al., 2022). The scFv of Rova, granzyme B and typhoid toxin were evaluated as controls along with immunotoxins. The scFv of Rova and granzyme B like immunotoxins were cloned, expressed and purified.

### 2.2 Cell culture

The human A549 lung cancer cell lines and the normal HUVEC, Human umbilical vein endothelial cell line, were purchased from the Pasteur Institute and Iranian Biological Resource Center (IBRC), respectively. DMEM high glucose and DMEM/F12 media containing 10% FBS and 1% Penicillin-Streptomycin used for cell culture and proliferation of A549 and HUVEC cell lines, respectively. The cells were passaged using 0.25% Trypsin-EDTA at around 80% confluence. Cells were seeded in 96-well plates at a density of 8 × 103 cells/well for the MTT assays. According to the studies, the A549 cell line is considered the DLL3 positive (Pancewicz et al., 2018) and the HUVEC cell line is DLL3 negative. All equipment, media and solutions were purchased from Bio-IDEA Company, IRAN.

### 2.3 Binding assay

In the first step, in order to confirm the expression of the DLL3 in A549 cell line, the ability of the scFv of Rovalpituzumab to bind to the DLL3 was evaluated by cell-ELISA method (Rezaie et al., 2020). For this purpose, 20,000 cells per wells were cultured in the ELISA plate and incubated for 16 h in CO2 incubator at 37°C. After washing with PBS buffer (phosphate-buffered saline), the A549 cells were fixed with 8% formaldehyde (Ghatran Chimi Tajhiz, Iran). The blocking step was performed using 7% bovine serum albumin (BSA) and incubation for 2 h. In the following, the cells were incubated with 2 nmol, 4 nmol, 10 nmol, 20 nmol, 30 nmol, 40 nmol, 120 nmol, 200 nmol, 400 nmol, and 1 and 2 µmol scFv of Rovalpituzumab for 1 hour. After washing 5 times with PBST buffer (PBS +0.05% Tween-20), to confirm the presence of the DLL3 on the surface of A549 cell line, HRP-conjugated anti-his tag antibodies (Sigma Aldrich, United States) and its substrate, TMB (3,3′,5,5′-Tetramethylbenzidine), was used. After 20 min, the reaction was stopped with 2 M sulfuric acid and the absorbance rate was measured at 450 nm using a microplate reader xMark model (Bio-Rad, Hercules, CA, United States). Next, the binding ability of immunotoxins to the DLL3 was determined by the Cell-ELISA method as mentioned above. Each concentration was repeated three times.

### 2.4 Cell viability assay

The toxicity of Rova-GrB, Rova-Typh was assessed after 24, 48 and 72 h by MTT assay kit (Bio-IDEA, Iran) (Rezaie et al., 2020). 8000 cells of A549 (DLL3 positive) and HUVEC (DLL3 negative) cell lines were cultured in 96 well plate for overnight, then incubated with different concentrations of Rova-GrB and Rova-Typh (15 nmol, 75 nmol, 150 nmol, 350 nmol, 700 nmol and 1, 1.5 µmol) for 24, 48 and 72 h at 37°C in a humidified atmosphere of 95% air and 5% CO2 incubator. 10 μL of MTT reagent (3-(4,5-Dimethylthiazol-2-yl)-2,5-diphenyltetrazolium bromide, 5 mg/mL) was added to each well and incubated in the same conditions for 4 h. Finally, the cell supernatant was discarded and 100 µL of DMSO (dimethyl sulfoxide, Neutron Pharmacheminal company, IRAN) was added to each well. Then, the intensity of the color produced, which indicates living cells, was read by the microplate reader (Bio-Rad, Hercules, CA, United States) at a wavelength of 570 nm. Each concentration was carried out in triplicate. It should be noted that the toxicity of the scFv of Rova, typhoid toxin and GrB alone was evaluated. It should be noted that each concentration was carried out in triplicate.

### 2.5 Apoptosis assay

In order to assess the level of apoptosis induction by Rova-GrB and Rova-Typh, the flow cytometry and annexin-PI staining method were utilized. A total of 200,000 cells were cultured in each well of 6-well ELISA plates. After 24 h, cells were treated with Rova-GrB )150 nmol, 350 nmol, 700 nmol( and Rova-Typh (350 nmol, 700 nmol, and 1 µmol), for an overnight. Subsequently, the wells were washed three times with 1X PBS and, the cells were detached by trypsin the cells were centrifuged at 500 g for 3 min at 4°C, and the supernatant was discarded. Then, 500 µL of DMEM growth medium was added to the cells. The cells were suspended in Anexin V Binding buffer and treated with 5 µL of Anexin FITC and propidium iodide stain. After 30 min of incubation and washing with cold 1X PBS, analysis was conducted using a flow cytometry instrument (FACS Calibur, Becton Dickinson, United States). Subsequently, the samples were sent to Saba Laboratory for analysis using the flow cytometry instrument. At this stage, each concentration was tested in triplicate times.

### 2.6 Penetration assay

To evaluate the penetration of Rova-GrB and Rova-Typh into A549 cell line, the Western blotting method was employed (Kurien and Scofield, 2015). A total of 200,000 cells were cultured in each well of 6-well plates. After 24 h, cells were treated with 350 nmol of Rova-GrB and 700 nmol of Rova-Typh for one, two, and 3 hours at 37°C. Following incubation, to remove unbound immunotoxins, cells were washed three times with 1X PBS. Subsequently, cells from each well were detached using trypsin, and centrifuged (similar to Section 2.5. Flow Cytometry Apoptosis Assay). Next, cells were lysed using RIPA buffer (50 m*M* Tris–HCl (pH 7.4), 150 m*M* NaCl, 1% Triton X-100, 0.5% sodium deoxycholate, 0.1% SDS, and 5 m*M* EDTA), and the samples were loaded onto a 12% SDS-Page gel. Considering that the recombinant immunotoxins possess a polyhistidine-taqs, the Western blotting method was employed to confirm the entry of immunotoxins into A549 and HUVEC cell lines. In this method, 10 µL of the sediment of A549 cell line treated with immunotoxins during different hours, which was lysed by RIPA buffer, was loaded on a 12% SDS-Page gel and electrophoresis was performed. Next, the proteins were transferred from the gel to the PVDF paper. After the blocking and washing steps, the presence of Rova-GrB and Rova-Typh was evaluated using mouse anti-his tag antibody HRP conjugated.

### 2.7 Statistical analysis

All data are expressed as mean ± standard deviation. The one-way analysis of variance (ANOVA) was used where appropriate for statistical analysis by SPSS software. All tests were two-sided and p < 0.05 was considered significant. Each concentration was carried out in triplicate.

## 3 Results

### 3.1 Confirmation of DLL3 presence on the surface of A549 cell

**FIGURE 3:**
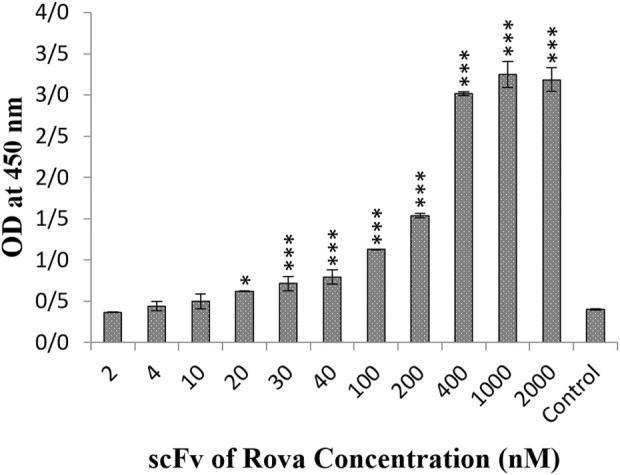
The result of the binding of scFv of rovalpituzumab to the DLL3 receptor on the surface of the A549 cell line. The control group is without scFv of Rova (OD, optical density). The significance of the results (p-Value) between the control group and other groups has been indicated as follows: *P < 0.05; **P < 0.01; **P < 0.001.

The results obtained from the Cell-ELISA method indicated that DLL3 is well expressed in the A549 cell line, saturating at concentrations exceeding 400 nmol of the scFv of rovalpituzumab antibody (Figure 3). At concentrations of 40 nmol, 120 nmol, 200 nmol, 400 nmol and 1 μmol, the scFv of Rova antibody bound to the DLL3 receptor on the A549 cell surface by 14.4%, 25.3%, 40%, 92%, and 100%, respectively, compared to the negative control group.

### 3.2 Immunotoxin binding capability to DLL3 by the cell-ELISA method

**FIGURE 4:**
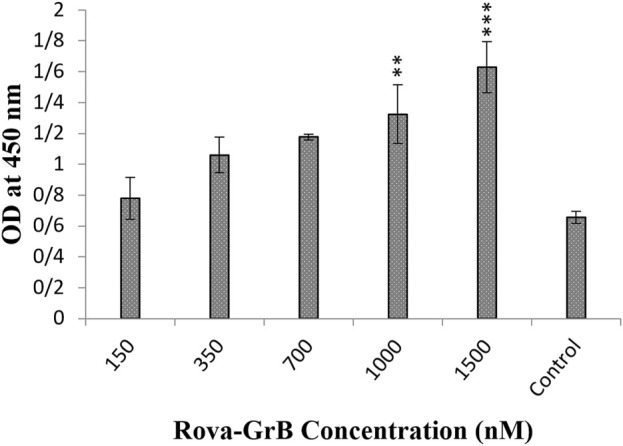
Evaluation of the binding ability of recombinant immunotoxin Rova-GrB on the A549 cancer cell line using the Cell-ELISA method (The control group is without Rova-GrB). The significance of the results (p-Value) between the control group and other groups has been indicated as follows: *P < 0.05; **P < 0.01; **P < 0.001.

After 1 hour of incubation of immunotoxins Rova-GrB and Rova-Typh with A549 cells, the results showed that the binding strength of Rova-GrB at concentrations of 350, 700, 1000 and 1500 nmol to the DLL3 receptor increased by 30%, 44%, 66.6% and 100% respectively compared to the control group (Figure 4).

**FIGURE 5:**
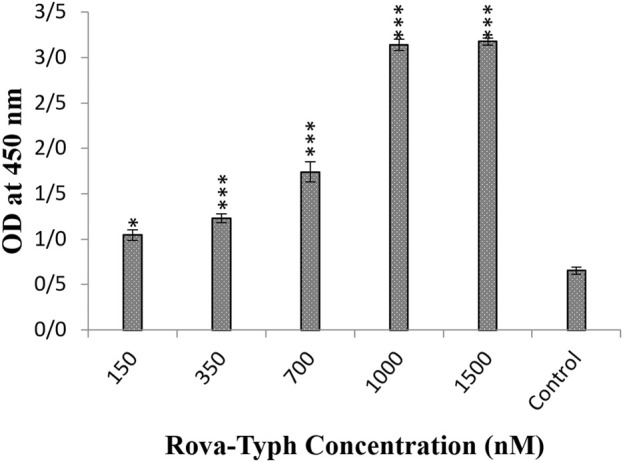
Evaluation of the binding ability of recombinant immunotoxin Rova-Typh on the A549 cancer cell line using the Cell-ELISA method (The control group is without Rova-Typh). The significance of the results (p-Value) between the control group and other groups has been indicated as follows: *P < 0.05; **P < 0.01; **P < 0.001.

Furthermore, the results demonstrated that the Rova-Typh at concentrations of 150, 350, 700, 1,000, and 1,500 nmol, bound to the DLL3 receptor on the surface of A549 cells by 7.4%, 15.5%, 37%, 99%, and 100%, respectively compared to the control group (Figure 5).

**FIGURE 6:**
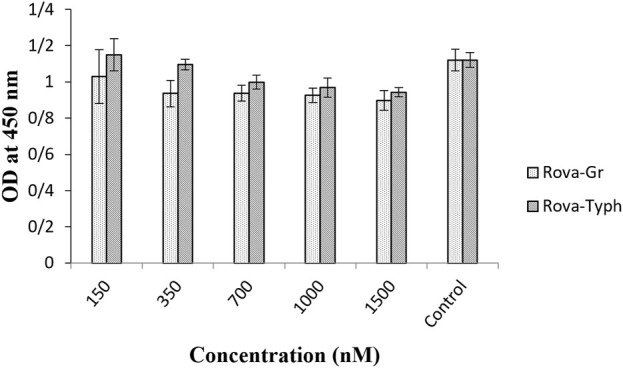
Evaluation of the binding ability of recombinant immunotoxins on the HUVEC normal cell line using the Cell-ELISA method (The control group is without Rova-GrB and Rova-Typh).

The results indicate that both immunotoxins can bind to the DLL3 receptor on the surface of cancer cells, while they do not exhibit the capability to bind to the surface of the HUVEC cell line (Figure 6).

### 3.3 Cytotoxic effects of immunotoxins and by the MTT assay

**FIGURE 7:**
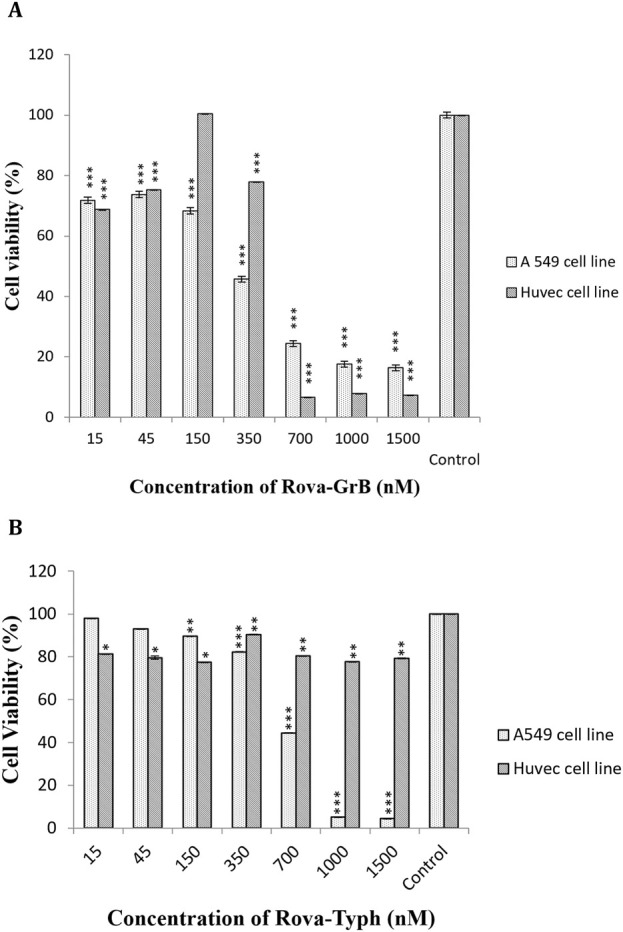
**(A, B)** The cell viability percentage of Rova-GrB and Rova-Typh on A549 and HUVEC cell lines after 24 h of incubation (The control group is without Rova-GrB and Rova-Typh). The significance of the results (p-Value) between the control group and other groups has been indicated as follows: *P < 0.05; **P < 0.01; **P < 0.001.

The cytotoxic effects of immunotoxins were evaluated on A549 and HUVEC cells using the MTT assay during 24, 48, and 72 h of incubation at 37°C. The results demonstrated a statistically significant difference in cytotoxicity with increasing concentrations of Rova-GrB and Rova-Typh (p-value <0.01) in A549 cells, specifically at concentrations higher than 338 and 734 nmol, respectively. In fact, the immunotoxins effectively eliminated A549 cancer cells at a concentration gradient. The IC50 values for Rova-GrB and Rova-Typh immunotoxins at 24 h were 338 and 734 nmol, respectively (The IC50 value of the immunotoxins were obtained by using Excel software). Notably, while Rova-Typh immunotoxin did not have a significant cytotoxic effect on the HUVEC normal cell line, Rova-GrB immunotoxin exhibited a meaningful cytotoxic effect at concentrations of 700 nmol and higher on the HUVEC normal cell line (see Figures 7A, B).

**FIGURE 8:**
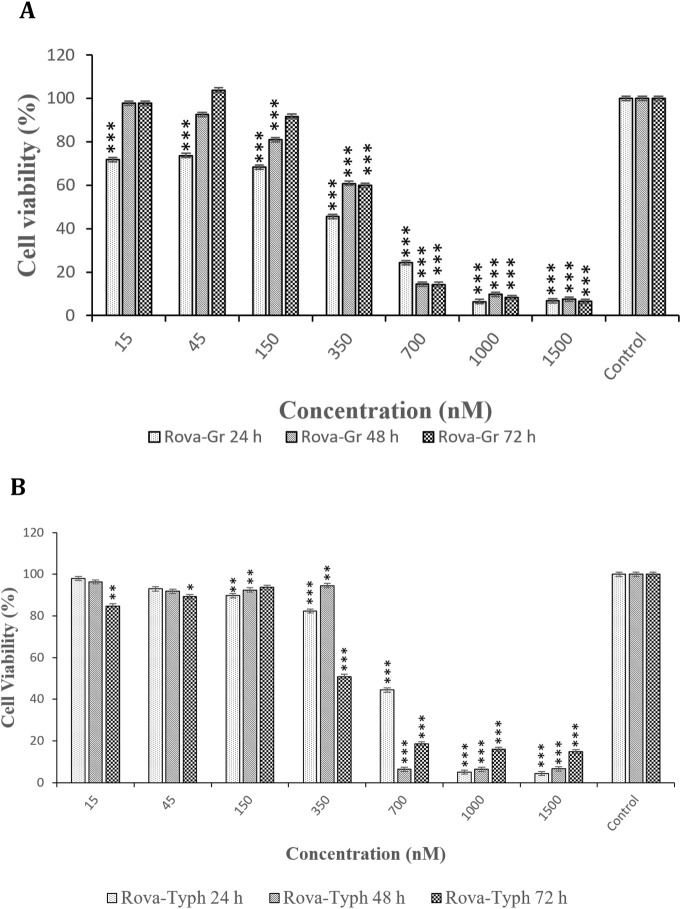
**(A, B)** Comparison of the cytotoxic effects of Rova-GrB and Rova-Typh at 24, 48, and 72 h on A549 lung cancer cell line (The control group is without Rova-GrB and Rova-Typh). The significance of the results (p-Value) between the control group and other groups has been indicated as follows: *P < 0.05; **P < 0.01; **P < 0.001.

The assessment of cytotoxic effects of immunotoxins at different time points (24, 48, and 72 h) on the A549 cell line indicated that the cytotoxic effects of both immunotoxins at 48 and 72 h were similar to those at 24 h, with no significant difference (see Figures 8A, B).

**FIGURE 9:**
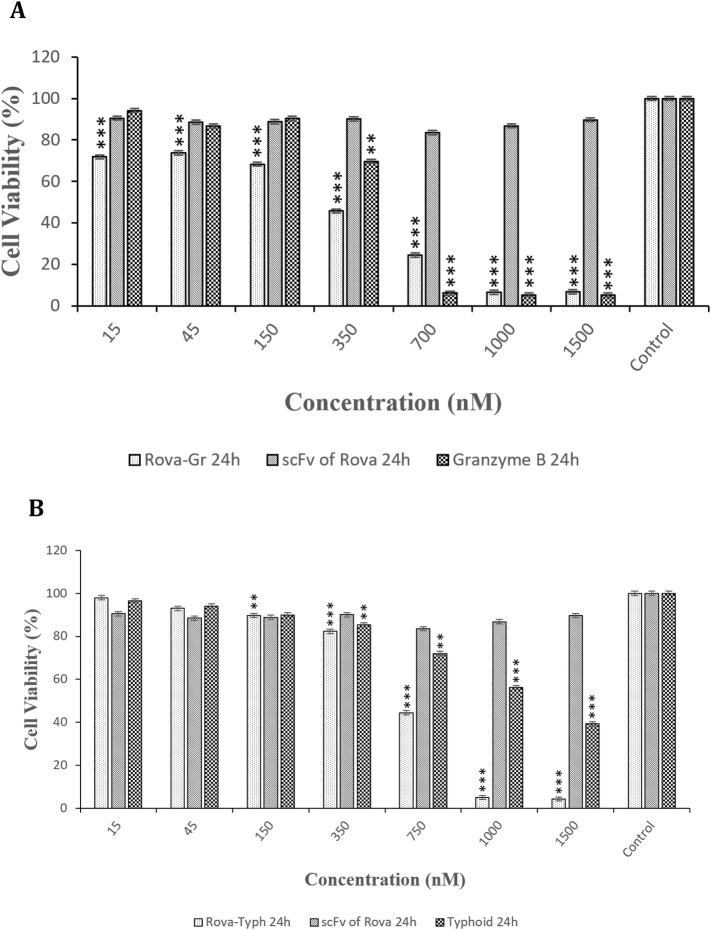
**(A, B)** Comparison of the cytotoxic effects of Rova-GrB, Rova-Typh, scFv of Rova, Granzyme B, and Typhoid Toxin on A549 lung cancer cell line after 24 h of incubation (The control group is without Rova-GrB, Rova-Typh, scFv of Rova, Granzyme B, and Typhoid Toxin). The significance of the results (p-Value) between the control group and other groups has been indicated as follows: *P < 0.05; **P < 0.01; **P < 0.001.

Additionally, the effects of immunotoxins and their components, as a control, namely, scFv of Rova, Granzyme B, and Typhoid Toxin, were evaluated individually over 24 h on the A549 lung cancer cell line. The results from the effects of the immunotoxin components showed that scFv of Rova alone had no cytotoxic effects. However, Granzyme B exhibited significant cytotoxic effects at concentrations of 700 nmol and higher, while Typhoid Toxin, when tested alone, displayed lower cytotoxic effects compared to Granzyme B (Figures 9A, B).

### 3.4 Apoptosis

**FIGURE 10:**
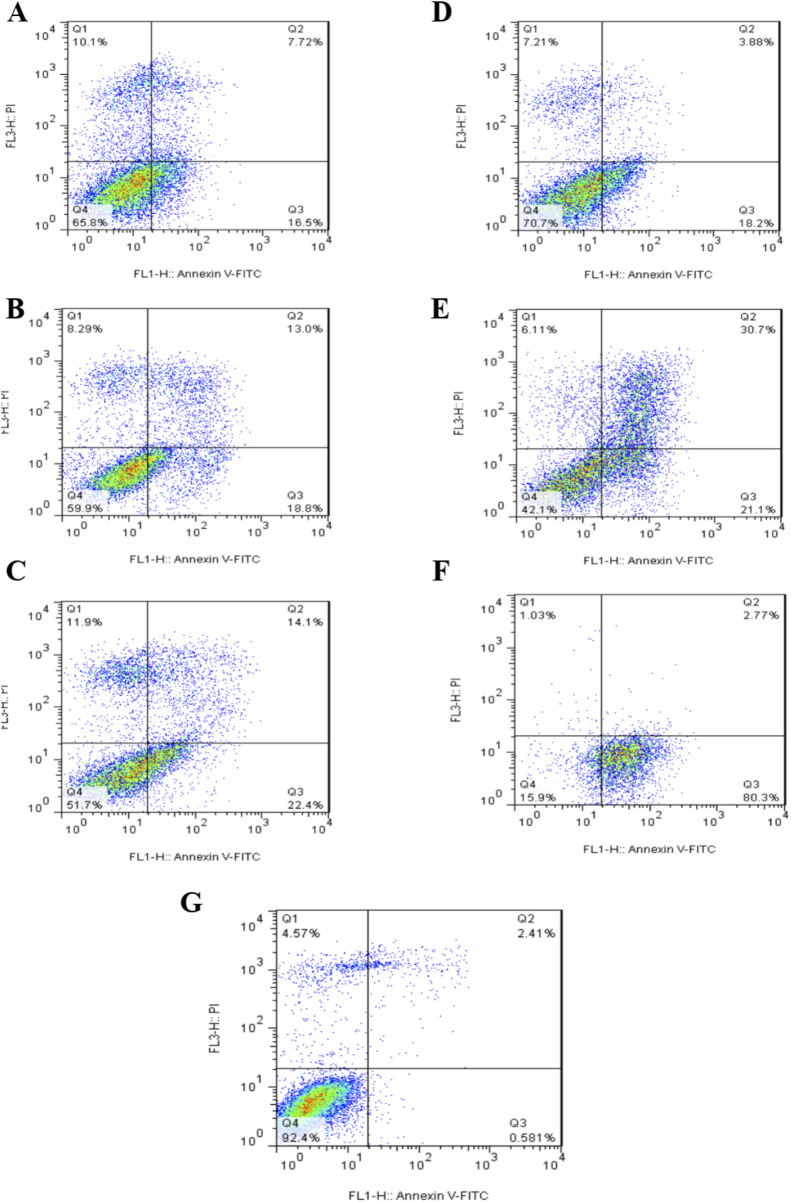
Assessment of the level of programmed cell death (apoptosis) in the A549 lung cancer cell line at different concentrations of immunotoxins. Assessment of the level of programmed cell death (apoptosis) and necrosis in A549 cells treated with Rova-GrB at a concentration of 150 nmol **(A)**, 350 nmol **(B)** and 700 nmol **(C)**. Assessment of the level of programmed cell death (apoptosis) and necrosis in A549 cells treated with Rova- Typh at a concentration of 350 nmol **(D)**, 700 nmol **(E)** and 1,000 nmol **(F)**. Control cells treated with PBS **(G)**. Representative figures showing the population of viable (Q4), early apoptotic (Q3), late apoptotic (Q2) and necrotic (Q1) cells.

The results of immunotoxin-induced apoptosis were assessed using the PI/Annexin-V staining method, and cell death was measured using flow cytometry. Figure 10, Table 1 illustrates the type of cell death, including necrotic cells, late apoptosis, early apoptosis, and normal cells. A549 cells were treated with concentrations of 150 nm, 350 nm, and 700 nmol of Rova-GrB immunotoxin and concentrations of 350 nm, 700 nm, and 1000 nmol of Rova-Typh immunotoxin. A concentration of 2,000 nmol of the recombinant protein scFv of Rova was used as a negative control due to its lack of the catalytic domain (Not shown). The percentage of apoptosis (both early and late) in A549 cells treated with Rova-GrB and Rova-Typh was determined to be 31.8% and 51.8%, respectively. Furthermore, a significant difference was observed in the increase in total apoptosis (both early and late) in the A549 lung cancer cell line. These results indicate the potency of the immunotoxins in inducing programmed cell death, making them promising candidates for targeted drug development.

**TABLE 1:** Detail of apoptosis data for recombinant immunotoxins and components.

	Rova- GrB			Rova-Typh			GrB			scFv of rovalpituzumab	Negative control without treatment
Percentage of Cells	150 nMol	350 nMol	700 nMol	350 nMol	700 nMol	1 µMol	150 nMol	350 nMol	700 nMol	700 nMol	-
Q1	10.1	8.29	11.9	7.21	8.60	1.03	3.74	6.47	3.40	3.94	4.57
Q2	7.72	13	14.1	3.88	34.9	2.77	19.9	31.6	63.8	4.35	2.41
Q3	16.5	18.8	22.4	18.2	15.4	80.3	43.5	33.1	29	3.43	0.5
Q4	65.8	59.9	51.7	70.7	41.1	15.9	32.9	28.9	3.89	88.3	92.4

### 3.5 Immunotoxin permeability

**FIGURE 11:**
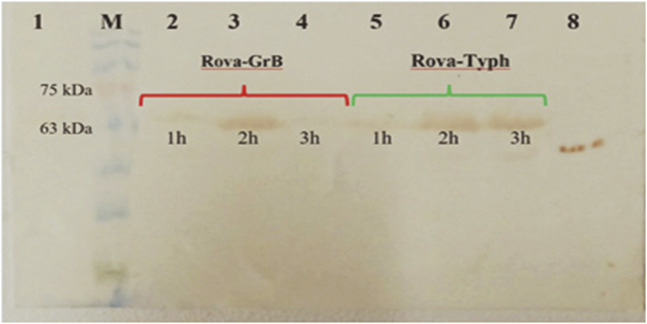
Examination of the penetration of immunotoxins into A549 lung cancer cells. 1) Untreated A549 cancer cells (negative control). 2) A549 cell lysate after 1 h of treatment with Rova-GrB. 3) A549 cell lysate after 2 h of treatment with Rova-GrB. 4) A549 cell lysate after 3 h of treatment with Rova-GrB. 5) A549 cell lysate after 1 h of treatment with Rova-Typh. 6) A549 cell lysate after 2 h of treatment with Rova-Typh. 7) A549 cell lysate after 3 h of treatment with Rova-Typh. 8) Positive control. M) Molecular weight marker protein, SL7011, SINACLON Co.

The Western blotting method was used to assess the entry of recombinant immunotoxins into the A549 lung cancer cell line. In each well of a 24-well cell culture plate, approximately 2 × 10^5^ cells were seeded and incubated for 24 h. Subsequently, concentrations of 350 nmol and 700 nmol of Rova-GrB and Rova-Typh, respectively, which were determined as the IC50 in the previous stages, were used. The permeability of immunotoxins at each of the specified concentrations was assessed at 1, 2, and 3 h (Figure 11). The Western blot results indicate that the highest level of entry of Rova-GrB was observed after 2 h, while Rova-Typh showed the highest entry after 3 h on the PVDF membrane.

## 4 Discussion

The design and development of immunotoxins against SCLC have been underway for over 34 years since the beginning of research by Weltman and colleagues in 1987. Specific biomarkers on cancer cells play a crucial role in the identification, progression, prevention, and treatment of cancer. Scientists and researchers have made significant efforts to identify specific biomarkers in primary tumors and metastatic cancer forms. These aims, to prevent disease progression by identifying these biomarkers in primary tumors and to treat the disease in the metastatic form by targeting these biomarkers with targeted drugs, were taken into account. Therefore, the identification of new biomarkers that can be used for the detection of both primary and metastatic lung cancer tumors is essential. Overexpressed antigens are considered suitable candidates for targeted delivery to cancer cells (Mir and Islam, 2016).

Receptors selected as targets for immunotoxins should exhibit increased expression at the surface of cancer cells compared to normal cells. DLL3, chosen in this study, is among the inhibitory ligands of the NOTCH signaling pathway. It is expressed in neuroendocrine and SCLC tumors, while it is not expressed in normal tissues. The 80% expression of DLL3 on SCLC lung cancer cells makes it a suitable target for treatment with targeted drugs, including immunotoxins (Rojo et al., 2020). New treatments targeting DLL3, such as CAR-T, BiTE, TiTE, and CAR-NK, are currently being evaluated in various phases of clinical trials for patients with SCLC and show encouraging results. However, the use of immunotoxins aimed at targeting DLL3 has remained overlooked (Su et al., 2024).

In this study, it was determined that the scFv of Rova, when used alone at concentrations of 200 nmol or higher, can effectively bind to the DLL3 receptor on A549 cell line. These results confirm the findings of DLL3 expression on the surface of A549 cell line using Real-Time PCR, as reported by Pancewicz and colleagues in 2018. One implication of designing an immunotoxin based on bacterial toxins is the targeted binding to cancer cells, entry into these cells, and the selective destruction of cancer cells. However, it was observed that the GrB-based immunotoxin exhibits cytotoxic effects prior to entering the cell. Although GrB is a potent enzyme with multiple substrates and this study has shown that the GrB-based immunotoxin demonstrates better cytotoxic effects, it appears that due to its low binding affinity to the DLL3 receptor and its non-specific cytotoxic effects, it cannot be considered an ideal immunotoxin.

The results of the Cell-ELISA method demonstrated that both immunotoxins can bind to A549 cells while no attachment was observed with HUVEC cells. Furthermore, the Cell-ELISA results revealed that Rova-Typh has a stronger binding affinity to DLL3 on the surface of A549 cell lines compared to Rova-GrB (Figures 4, 5). In other words, the produced immunotoxin can exhibit highly specific binding to cancer cells.

It appears that structural changes in scFv of Rova induced by Granzyme B and PltA of typhoid toxin have caused the third structure of Rova-Typh to be more extended, likely contributing to the stronger binding of Rova-Typh to DLL3. In other words, scFv of Rova and Rova-Typh are both capable of complete binding to the DLL3 receptor at 400 and 150 nmol concentrations (saturation concentration) respectively, whereas the binding capability of Rova-GrB to the receptor is significantly lower. The reason may lie in the third structure of the immunotoxins. The third structure of Rova-Typh is elongated and thin, with different domains of the immunotoxin clearly separated from each other, while the third structure of Rova-GrB is short and thick, and the compression of different domains may hinder its proper binding to the DLL3 receptor (Ataee et al., 2022).

In the present study, the cytotoxic effects of the immunotoxins Rova-GrB and Rova-Typh on A549 cells expressing the DLL3 receptor were investigated using the MTT assay. This method is based on the reduction of tetrazolium salt to formazan crystals by mitochondrial enzymes. The results indicated that A549 cells exhibit a high sensitivity to immunotoxins after 24 h of incubation. This sensitivity is directly correlated with the concentration of immunotoxins, as an increase in the immunotoxin concentration resulted in higher levels of apoptosis and, ultimately, cell death in cancer cells. In general, it can be concluded that the cytotoxicity of this cell line is dependent on the concentration of immunotoxins.

Granzyme B is structurally similar to serine proteases and activates caspase 3, and directly by breaking Bid and ICAD proteins, it activates the damage pathway to mitochondria and DNA. Mitochondria damage by Granzyme B causes the production of ROS, the release of cytochrome C and other pro-apoptotic molecules in the mitochondria and finally causes cell death. Also, the presence of Granzyme B outside the cell causes damage to the extracellular matrix (vitronectin, fibronectin and laminin), its proteins and proteolysis of cell surface receptors, cutting of Notch1, FGFR1 and GPCR proteins (Chowdhury and Lieberman, 2008).

Typhoid toxin is a toxin with A2B5 structure, subunit B is responsible for binding and two catalytic subunits, one with nuclease property (PltB) and the other with ADP-ribosylation property (PltA), also PltA in terms of amino acid sequence, it is completely similar to pertussis toxin S1. This toxin binds to NAD+ in the cytoplasm of the cell and ADP-ribosylates G proteins and then increases the concentration of cAMP and subsequently by activating protein kinase A, it causes to stop or activate the cell cycle, which depends on various factors. There are several signaling pathways downstream of PKA that lead to cell death. Although the role of typhoid toxin cytotoxicity points to the simultaneous action of PltA and PltB, the substrates of PltA have not yet been fully elucidated (Cheng and Wiedmann, 2019; Chong et al., 2017; Simon et al., 2014; New and Wong, 2007; Persaud et al., 2018).

Granzyme B has more substrates against typhoid toxin, so it is expected to show more cytotoxicity effects in a non-targeted manner.

In contrast, HUVEC cells showed no sensitivity at IC50 concentrations of the immunotoxins. Comparing the IC50 values between the immunotoxins, it can be observed that Rova-GrB is approximately twice as toxic as Rova-Typh. The reason for this difference depends on the catalytic domain of the immunotoxins. Regarding the cytotoxic effects of the catalytic domain, GrB and PltA of Typhoid Toxin alone were observed to induce cell death within 24 h. GrB at concentrations of 2 μmol and higher caused more than 90% cell death, while PltA of Typhoid Toxin at a concentration of 3.7 μmol induced approximately 60% cell death (Figures 9A, B). Therefore, it is expected that these catalytic portions behave similarly in the immunotoxin structures, and the results of the MTT assay confirm this hypothesis. Rova-GrB exhibits cytotoxic effects in the HUVEC cell line at concentrations of 700 nmol and above. These toxic effects are higher than the IC50 concentration of the immunotoxin in the A549 cell line, and the non-specific activity of GrB in the structure of the immunotoxin at the cellular surface may be the cause.

In contrast, in the HUVEC cell line, which lacks DLL3 expression, Rova-Typh had no cytotoxic effects, while Rova-GrB exhibited cytotoxic effects at concentrations of 700 nmol and higher. Although there have been no reports on the design and application of DLL3 receptor-based immunotoxins for SCLC treatment so far, Mattoo and colleagues utilized a combination of a chemical drug called navitoclax (a BCL-2 inhibitor) and an immunotoxin targeting the transferrin receptor called HB21-PE40 for SCLC treatment. The research results indicate that neither of these drugs has therapeutic effects when used individually, but the combination of these drugs, specifically HB21-PE40 at 10 ng/mL + ABT 263 (3 μM), leads to complete cell death in SCLC cell lines after 48 h.

Ehrlich and colleagues developed an immunotoxin named BW-2, consisting of an antibody against a RNA-binding protein in neurons called HuD and the toxin saporin, for the treatment of SCLC and neuroblastoma in HuD-expressing cancer cells and tumor models. The results showed that the IC50 of the immunotoxin BW-2 after 72 h is 0.5 μg/mL. In the most recent study, an immunotoxin composed of an anti-Glypican-3 (GPC3) antibody and *Pseudomonas aeruginosa* exotoxin A (PE38) was designed for the treatment of SCLC and liver cancer. The results indicated that the IC50 of the immunotoxin hGC33 PE38 after 48 h in the lung cancer cell line H466 is 71 ng/mL. Cytotoxicity data at 24 h were not reported in this study. Considering the conducted research, it appears that the difference in IC50 concentrations in our study compared to the cases mentioned depends on the level of DLL3 expression in the SCLC cell lines used in different studies. Furthermore, despite the expectation that the IC50 of the immunotoxin Rova-Typh would be higher than Rova-GrB based on the results of the Binding assay, the actual results were the opposite. The reason for this may be the non-specific activity of Granzyme B in the structure of the Rova-GrB immunotoxin. Our results showed that within 24 h, the cytotoxic effect of GrB alone at a concentration equivalent to the Rova-GrB immunotoxin, 50 μg/mL, is higher. Additionally, at higher concentrations, the cytotoxic effects of the Rova-GrB immunotoxin and GrB alone are similar. Given that the isoelectric point of Granzyme B is around 10 and it carries a positive surface charge, it can non-specifically bind to proteins that have a negative surface charge on the cell surface, such as glycosaminoglycans, gangliosides, and sulfated lipids, and enter cells through endocytosis to exert its catalytic effects. According to our studies, cancer cell surface charge is strongly negative. Therefore, it can be said that GrB can non-specifically bind to the surface of A549 cells at concentrations higher than 2 µmol and exhibit toxic effects. In other words, Granzyme B is a potent serine protease known to recognize over 58 substrates, some of which are located in the nucleus, some in the cytoplasm, and some on the plasma membrane. Although our expectation for the designed immunotoxin was that it would demonstrate its function after entering the cell and releasing the catalytic portion in the cytoplasm, the results of the cytotoxic effects of recombinant immunotoxin rRova-GrB indicate that Granzyme B in the structure of the immunotoxin is also an active enzyme before entering the cell, in a way that it can cleave membrane proteins at the site of aspartic acid peptide bond even on the cell surface. Therefore, the IC50 of the immunotoxin rRova-GrB was approximately half of that of the immunotoxin rRova-Typh.

Overall, it was evident that the designed immunotoxins possess significant potential for killing DLL3-expressing cells. Interestingly, while based on the MTT results, we expected Rova-GrB to induce higher levels of apoptosis compared to Rova-Typh, in practice, Rova-Typh exhibited much stronger apoptosis induction. This could be attributed to the binding affinity of these immunotoxins. The binding assay revealed that Rova-Typh had twice the binding affinity to the DLL3 receptor on A549 cancer cells compared to Rova-GrB. Therefore, it is likely that a higher amount of this immunotoxin was able to enter the cancer cell cytoplasm and induce apoptosis.

Apoptotic cell analysis is performed using the Annexin V-FITC/PI staining method and flow cytometry technique. In this method, cells undergoing apoptosis are stained with FITC-conjugated Annexin due to the presence of phosphatidylserine on their surface, making them countable. In contrast, cells that have undergone necrosis are stained with propidium iodide (PI). Notably, approximately 50% of apoptosis induction by Rova-Typh was observed precisely at its IC50 concentration, whereas Rova-GrB at this concentration only exhibited 36% apoptosis induction.

In other words, the results from the binding assay showed that rRova-Typh exhibits a higher affinity for the DLL3 receptor on the surface of cancer cells. As shown in Figure 5, rRova-Typh reached saturation by binding to all DLL3 receptors on the surface of cancer cells at a concentration of 150 nmol. Meanwhile, rRova-GrB bound to only half of the DLL3 receptors on the cell surface at a concentration of 2 μmol. The results of the immunotoxin binding assessment phase fully support the apoptotic outcomes. Additionally, considering the results of the interaction prediction between immunotoxins and the DLL3 receptor using the ClusPro online server, it was determined that the stability of the Rova-Typh binding (−1800 KJ/Mol) is higher than that of Rova-GrB (−1400 KJ/Mol) against the receptor (unpublished data). Therefore, it is expected that the binding of Rova-Typh and its subsequent penetration into cancer cells will be more pronounced. The laboratory experimental results (binding assay) corroborate the findings from the bioinformatics prediction phase.

The results (of our old and new study) have significantly validated the bioinformatics methods, and this study suggests that the use of bioinformatics approaches can greatly reduce experimental work, particularly in identifying the structure and function of recombinant proteins as potential therapeutic candidates. It appears that adding a flexible and versatile linker (G4S)3 to the immunotoxins can greatly assist in enhancing their structural flexibility and overcoming physical hindrance to their dual functional roles. The designed immunotoxin, Rova-Typh, demonstrates effective expression and binding to DLL3-expressing cells. Furthermore, the results indicate that this immunotoxin, after binding, can efficiently undergo endocytosis into cancer cells and subsequently induce apoptosis, effectively eliminating DLL3-expressing cancer cells within 24 h. Therefore, these immunotoxins could have the potential to be suitable drug candidates for the treatment of SCLC lung cancer.

In future research, it is essential to investigate the effects of immunotoxins in animal models of tumors and other DLL3-expressing cancer cells, such as skin cancer, neuroblastoma, etc., to provide more precise insights into the anticancer effects of immunotoxins.

## 5 Conclusion

The latest studies to date reveal a growing interest among researchers in cancer treatment using targeted therapeutic approaches. Currently, 12 immunotoxins are registered and in various phases of clinical trials on the clinical trial website https://clinicaltrials.gov/. The targeted delivery of immunotoxins to cancer biomarkers, their efficient intracellular trafficking, and minimal impact on normal cells are among the most significant advantages of immunotoxins. However, the immunogenicity of immunotoxins and the production of anti-drug antibodies in the human body remain important limiting factors in their use. The precise design of immunotoxins in all three domains: targeting, translocation, and toxicity, such that it preserves and enhances the function of both domains while minimizing the size of each component, is expected to improve the performance of immunotoxins. In general, this research demonstrates that the use of immunotoxins induces significant cytotoxic effects in lung cancer cell lines. This study is a preliminary evaluation and development of immunotoxins. Evaluating immunotoxins in animal models and optimizing their structure to reduce immunogenicity and side effects can significantly enhance their performance. In subsequent phases (animal model), more detailed clinical aspects will be investigated.

## Data Availability

The datasets presented in this study can be found in online repositories. The names of the repository/repositories and accession number(s) can be found in the article/Supplementary Material.
