# Supplementary material for: Comparison of two immunotoxins against DLL3 receptor; as an inhibitor for small cell lung cancer
Source: Front Mol Biosci. 2025 Mar 19;12:1506768. doi: 10.3389/fmolb.2025.1506768 (PMC11963733; doi:10.3389/fmolb.2025.1506768)
Supplement: Supplementary file 1 [file Table1.docx]

Supplementary Table 1. A summary of critical data.

| Rova-Typh | Rova-GrB |  |
| --- | --- | --- |
| 734 nMol | 338 nMol | IC50 |
| 40% | 30% | Binding assay at IC50 concentration |
| 51.8% | 31.8% | Apoptosis assay at IC50 concentration  (early and late) |
