# Supplementary material for: Comparison of two immunotoxins against DLL3 receptor; as an inhibitor for small cell lung cancer
Source: Front Mol Biosci. 2025 Mar 19;12:1506768. doi: 10.3389/fmolb.2025.1506768 (PMC11963733; doi:10.3389/fmolb.2025.1506768)
Supplement: Supplementary file 2 [file Image1.pdf]

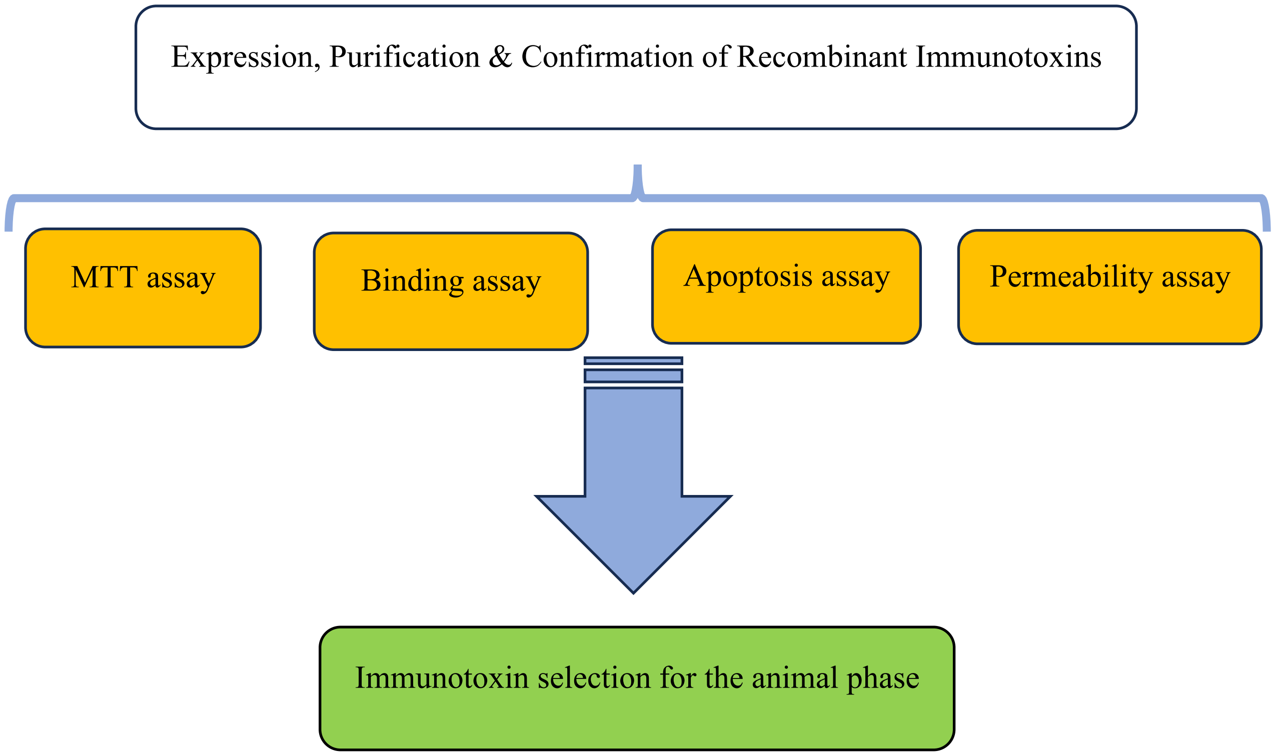
Expression, Purification & Confirmation of Recombinant Immunotoxins
MTT assay
Binding assay
Apoptosis assay
Permeability assay
Immunotoxin selection for the animal phase
